# Hypocalcemia in Elderly Population in a Tertiary Care Hospital: A Descriptive Cross-sectional Study

**DOI:** 10.31729/jnma.5324

**Published:** 2020-11-30

**Authors:** Sangita Thapa, Rabindra Jang Rayamajhi

**Affiliations:** 1 Department of Biochemistry, Kathmandu Medical College Teaching Hospital, Duwakot, Bhaktapur, Nepal; 2 Department of Internal medicine, Shree Birendra Hospital, Chhauni, Kathmandu, Nepal

**Keywords:** *elderly*, *hypocalcemia*, *serum calcium*

## Abstract

**Introduction:**

As the medical facilities are improving, the life expectancy is increasing which has led to rapid rise in elderly population. The epidemiology of many diseases in elderly has been modified, including calcium imbalance. This study aims to know the prevalence of hypocalcemia in elderly population visiting a tertiary care center of Kathmandu.

**Methods:**

A descriptive cross-sectional study was conducted in a tertiary care center of Kathmandu from March to July 2020 after obtaining ethical clearance (Ref: 2003202007). Total 402 participants at or above 60 years of age groups visiting outpatient departments were included in the study by convenience sampling method excluding those under vitamin D and calcium supplements. Serum total calcium level was measured using standard routine method and corrected with albumin. The serum calcium value less than 8 mg/dl was considered as hypocalcemia in accordance with the reference range of our laboratory. Data analysis for calculation of frequency and proportion was done in Statistical Package of Social Sciences.

**Results:**

The prevalence of hypocalcaemia in elderly was found to be 97 (24.1%). Out of 286 participants of age group 60-74 years, hypocalcemia was seen in 75 (26.2%) and among 116 participants of age group > 74 years, 22 (19%) were hypocalcemic. Among 181 male participants, 44 (24.3%) had hypocalcemia and out of 221 female participants, 53 (24%) had hypocalcemia.

**Conclusions:**

The finding of present study suggests that hypocalcemia is common among elderly which can be life threatening. Therefore, regular monitoring of serum calcium is recommended for this age group.

## INTRODUCTION

The life expectancy is increasing with improvements in medical facilities, which has modified the epidemiology of many diseases including calcium imbalance and requires attention to provide health necessity to elderly. The Nepali Senior Citizens Act defines elderly as an adult at or above 60 years of age.^1^

The normal functioning of many biological processes like muscle contraction, nerve transmission, coagulation and several enzymatic actions require calcium. Therefore, hypocalcemia is associated with a major threat to life which may occur due to decreased dietary uptake, reduced absorption, hypovitaminosis D and comorbidities like chronic kidney disease and parathyroid dysfunction in elderly.^2–5^

Elderly group comprises around 9% of the total population in Nepal and increased incidence of hypocalcemia is expected as this group of population is expected to increase to 11% by 2030.^6^ Therefore, this study aims to assess the prevalence of hypocalcemia in elderly population visiting a tertiary care center of Kathmandu.

## METHODS

This is a descriptive cross-sectional study conducted in Kathmandu Medical College Teaching Hospital (KMCTH) from March 2020 to July 2020 after obtaining ethical approval from Institutional Review Committee of KMCTH (Ref.: 2003202007). Participants visiting KMCTH outpatient departments who were at or above 60 years of age were included in the study after obtaining written informed consent. Convenience non-probability sampling technique was used to select the study participants. Elderly participants on vitamin D and calcium supplementation were excluded.

Sample size was calculated using the formula:

$$\begin{array}{ll} \text{N} & \text{= }{\text{Z}}^{\text{2}}\times \text{p}\times \text{q}/{\text{e}}^{\text{2}}\\ & \text{= }1.96^{\text{2}}\times 0.61\times 0.39/\text{0}{\text{.05}}^{\text{2}}\\ & \text{= }365 \end{array}$$

where,

n = required sample size,
Z = 1.96 at 95% Confidence Interval
p = prevalence, 61.31%^7^
q = 100-61.31% = 38.69%
e = margin of error, 5%

Adding 10% non-response rate, sample size was estimated to be 402.

Blood samples were routinely drawn by venipuncture and centrifuged to separate serum. Serum total calcium and serum albumin were estimated in HumaLyzer Primus semi-automated clinical chemistry analyzer using reagents supplied by Human Diagnostics. Serum total calcium level was measured based on o-cresolphthalein-complexone method and corrected with serum albumin level according to the formula: corrected serum calcium = measured calcium + [(4.1-albumin) × 0.8].^8,9^ Serum calcium value of 8-11 milligrams/deciliter (mg/ dl) was considered normal, value less than 8 mg/dl as hypocalcemia and value above 11 mg/dl as hypercalcemia in accordance with the reference range of our laboratory. Statistical analysis was done using Statistical Packages for Social Sciences version 16. Mean with standard deviation (SD) were calculated for quantitative data. Frequency and percentage were calculated for categorical data like age groups, gender and calcium groups.

## RESULTS

**Figure 1:**
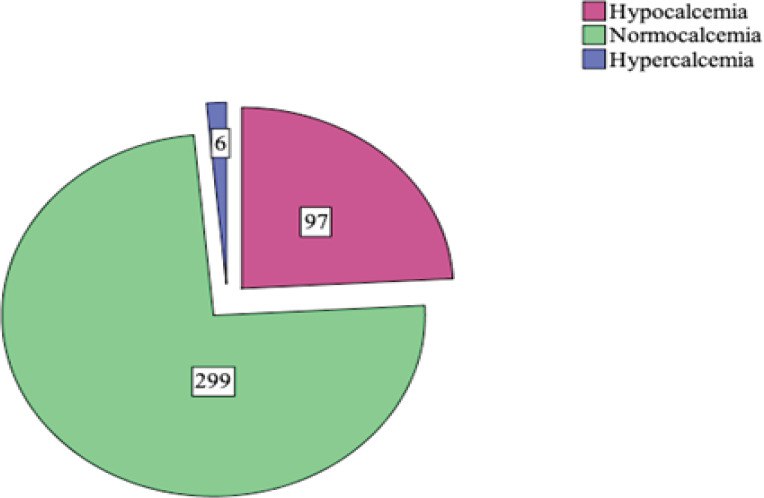
Distribution of total participants according to calcium status.

Out of 402 elderly participants, hypocalcaemia was found in 97 (24.1%) as depicted (Figure 1). Hypercalcemia was found among 6 (1.5%) participants and 299 (74.4%) had normal calcium level. The mean age of the hypocalcemic participants was 70.1±6.01 years.

Among total participants, 286 (71.1%) were in age group 60-74 years and 116 (28.9%) were >74 years of age. Hypocalcemia was found to be more in participants of 60-74 years of age 75 (26.2%) than the participants of >74 years of age 22 (19%) (Table 1).

**Table 1:** Distribution of serum calcium status according to age group.

Categories	Hypocalcemia n (%)	Normal calcium n (%)	Hypercalcemia n (%)
Total (n = 402)	97 (24.1)	299(74.4)	6 (1.5)
Age groups (years)			
60-74 (n = 286)	75 (26.2)	208 (72.7)	3 (1)
>74 (n = 116)	22 (19)	91 (78.4)	3 (2.6)

Out of total participants, 181 (45%) were male and 221 (55%) were female. Hypocalcemia was found to be slightly higher in male 44 (24.3%) than female 53 (24%) (Table 2).

**Table 2:** Distribution of Serum calcium status according to gender.

Gender	Hypocalcemia n (%)	Normal calcium n (%)	Hypercalcemia n (%)
Male (n = 181)	44 (24.3)	135 (74.6)	2 (1.1)
Female (n = 221	53 (24)	164 (74.2)	4 (1.8)

## DISCUSSION

The age related electrolytes deficiencies are common in elderly population. The present study was conducted to measure the prevalence of hypocalcemia among elderly and was found to be 24.1%. The mean age of participants with hypocalcemia was 70.1±6.01 years. The prevalence of hypocalcemia was seen more in 60-74 years of age than those above 74 years of age. Hypocalcemia is a multifactorial disorder and the studies on prevalence of hypocalcaemia in general population were very scanty for comparison. A study conducted by Catalano et al. reported that the prevalence of hypocalcemia among the participants over 65 years was 61.31% and in overall participants was 27.72%.^7^ Other studies, which compared the serum calcium between young and elderly group, have found lower serum calcium in elderly as compared to young participants.^10,11^

Serum calcium level is closely monitored and maintained by the action of parathyroid hormone, vitamin D and serum ionized calcium itself on kidney, bone and gastrointestinal tract.^12^ As ageing progresses, due to decreased dietary uptake and reduced absorption, calcium homeostasis is altered.^2^ Vitamin D deficiency, renal failure, and parathyroid hormone deficiency or resistances are the common culprits of hypocalcemia in elderly.^3–5,13^ A study conducted by Suryanarayana et al. found the prevalence of vitamin D deficiency to be 56.3% among elderly ≥ 60 years of age.^14^ The activity of renal enzyme, 1-α hydroxylase, is responsible for formation of active vitamin D, which decreases with age leading to vitamin D deficiency and ultimately results hypocalcemia in elderly.^15^ Andong et al. have suggested that the prevalence of chronic kidney disease increases with age, which can explain hypocalcaemia in elderly.^16^ The finding of the present study is in lieu with the widespread interest ascribed to deficiency of vitamin D and increased prevalence of chronic kidney disease seen in elderly. According to the present study, hypocalcaemia was found to be slightly more in male than female and this finding is comparable to Mansoor et al. and Catalano et al. which also reported similar findings in their respective studies.^17^

This study has certain limitations. Estimation of ionized calcium is considered as the most accurate method. However, due to cost factor and unavailability in our set up, we opted for albumin corrected calcium measurement. The samples were recruited from a single tertiary center so the results obtained may not be generalized to whole Nepalese population.

## CONCLUSIONS

The present study concludes that hypocalcemia is a frequently observed electrolyte disorder among elderly population, which can be a major threat to life. Regular monitoring of serum calcium is therefore mandatory in elderly. Additionally, identifying the underlying etiology of hypocalcemia and treating the cause along with calcium supplementation is recommended.
